# The value of transthoracic echocardiography in the detection of extra-cardiac lesions

**DOI:** 10.1186/s12893-022-01519-w

**Published:** 2022-02-26

**Authors:** Lei Yan, Qinyun Ruan, Chaoyang Qu, Chunyan Huang, Liyun Fu

**Affiliations:** Department of Ultrasound, The First Affiliated Hospital of Fujian Medical University, 20# Chazhong Road, Fuzhou, 350005 China

**Keywords:** Transthoracic echocardiography, Extra-cardiac lesion, Lung cancer, Mediastinal mass

## Abstract

**Objective:**

Transthoracic echocardiography (TTE) is generally recognized as the top choice for detecting myocardial and cardiac cavity lesions. Sonographers mostly focus on myocardium, cardiac cavity and cardiac hemodynamics, whereas the abnormal extra-cardiac lesions are easily remain unrecognized. The aim of this study was to investigate the ultrasonic image features in abnormal extra-cardiac lesions and the value of TTE in the detection of extra-cardiac lesions.

**Methods:**

49 cases of abnormal extra-cardiac lesion detected by TTE from January 2014 to December 2019 were collected, which were confirmed by surgical pathology. The two-dimensional ultrasonic characteristics and the relationships with the cardiac and great vessels were summarized on the basis of multi-view by TTE. All patients were also examined by computed tomography (CT).

**Results:**

In 49 patients with abnormal extra-cardiac lesions, 37 malignant cases and 12 benign cases were included. There were 41 cases (41/49, 86.67%) of mediastinal lesions and 8 cases (8/49, 16.33%) of lung lesions. The maximum diameter ranged from 3.2 cm to 13.66 cm, and the median diameter was about 7.4 cm, among which 29 cases (29/49, 59.18%) were larger than 5 cm. 4 cases (4/49, 8.16%) of cystic anechoic lesions were pericardial cyst. 2 cases (2/49, 4.08%) of cystic-solid echogenic lesions were teratoma. The remaining 43 cases (43/49, 87.76%) presented as solid hypoechoic or heterogeneous masses. 6 cases compressed the heart and 21 cases encroached on the heart and vessels. Diagnosis coincidence rates of TTE and CT were respectively 77.55% and 93.88%, with a statistical difference (*p* = 0.012).

**Conclusion:**

Although the diagnostic coincidence rate of TTE is slightly lower than that of CT, TTE has certain diagnostic value for extra-cardiac lesions.

## Introduction

Due to the interference of gas reflection in the lung surrounding the cardiac, the tissue structure around the cardiac can not be displayed. Radiologic methods, including CT [1] and magnetic resonance imaging (MRI) [2, 3], have always been the most commonly used imaging methods for diagnosing mediastinal and lung masses. However, TTE is able to detect the abnormal extra-cardiac lesions once solid or liquid lesions appear in the surrounding tissue. 49 patients with abnormal extra-cardiac lesions confirmed by surgical pathology were assessed to explore the value of TTE in detecting abnormal extra-cardiac lesions.

## Materials and methods

This study was approved by the Ethics Committee of the First Affiliated Hospital of Fujian Medical University with a written informed patient consent. All patient-sensitive information was protected with full confidentiality and only used for the purposes of this study.

49 patients with abnormal extra-cardiac lesions confirmed by surgical pathology in our hospital from January 2014 to December 2019 were collected, including 11 females and 38 males, with the age range of 10 months to 83 years old and the median age of 61 years old.

A comprehensive TTE examination (Ultrasonic instrument: GE vivid7, Philips IE33 and Philips Epic Q7 with transducers 1.7–3.4 MHz, USA) was performed on all patients. Two-dimensional images were captured with a frame rate of at least 70 frames per second and were recorded for analysis. Images of parasternal, apical, suprasternal and subxiphoid views were acquired, as well as other nonstandard planes if necessary.

The location, size, morphologic feature, echogenicity and relationships with the heart and the adjacent vessels were determined by TTE.

All patients were also examined by computed tomography (CT).

SPSS version 17.0 software (IBM Corporation, Armonk, NY) was used for statistical analysis. Continuous variables were expressed as mean ± 1 SD. The coincidence rates between the two groups were compared by Chi-square test. P < 0 0.05 was statistically significant.

## Results

37 malignant cases (37/49, 75.51%) and 12 benign cases (12/49, 24.49%) were included in 49 patients of abnormal extra-cardiac lesions. The types of disease were as follows: 8 cases of pulmonary lesions including 7 cases of primary lung cancer and 1 case of sarcoma pulmonary metastasis, 14 cases of esophageal cancer, 9 cases of lymphoma, 5 cases of lymph node metastasis, 4 cases of pericardial cyst, 1 case of pericardial maligant mesothelioma, 3 cases of thymoma, 3 cases of teratoma, 1 case of lipoma and 1 case of hiatal hernia.

The mediastinal lesions (41/49, 83.67%) were divided into 3 sections by pericardium: 14 cases (14/49, 28.57%) in the anterior mediastinum, 9 cases (9/49, 18.36%) in the middle mediastinum, and 18 cases (18/49, 36.73%) in the posterior mediastinum (see Table 1 for details).

**Table 1:** The distribution of extracardial lesions in mediastinum

Lesion	n	Anterior mediastinum		Middle mediastinum		Posterior mediastinum	
		n	%	n	%	n	%
Lymph node metastasis	5	0	0	2	40%	3	60%
Lymphoma	9	6	66.67%	3	33.33%	0	0
Thymoma	3	3	100%	0	0	0	0
Teratoma	3	3	100%	0	0	0	0
Pericardial cyst	4	0	0	4	100%	0	0
Pericardial mesothelioma	1	0	0	1	100%	0	0
Lipoma	1	0	0	1	100	0	0
Hiatal hernia	1	0	0	0	0	1	100%
Esophageal cancer	14	0	0	0	0	14	100%
Total	41	12	34.15%	11	21.95%	18	43.90%

**Fig. 1:**
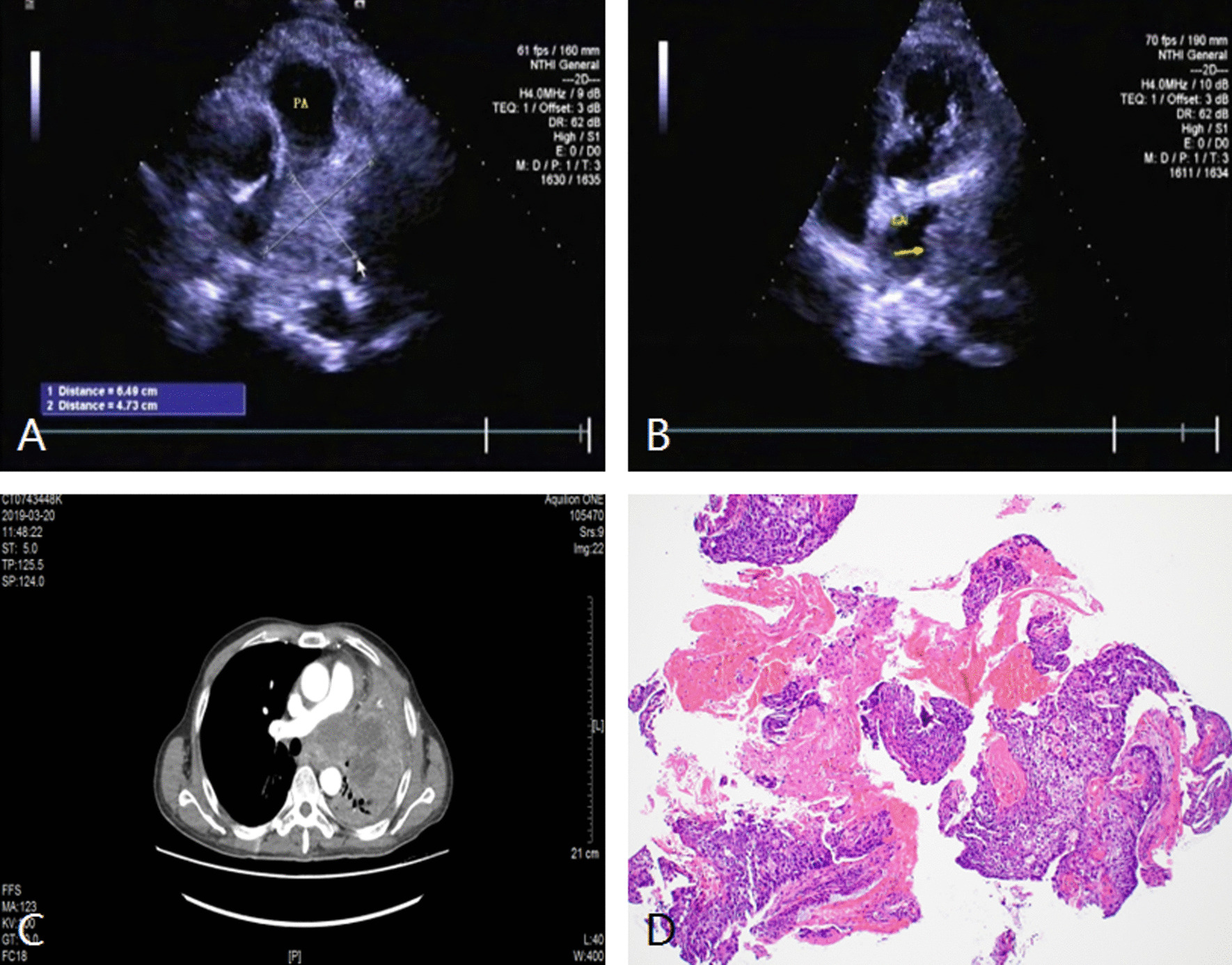
Left hilar lung cancer in a 51-year-old man. **A** and **B** TTE showed an irregularly shaped hyperechoic mass near the left hilum of the pulmonary artery bifurcation with left pulmonary artery and left pulmonary vein invasion. **C** Contrast-enhanced CT shows an irregularly shaped mass with mediastinal lymph node metastasis and obstructive atelectasis. **D** The pathologic diagnosis of this mass was moderately differentiated squamous cell carcinoma. TTE indicates transthoracic echocardiography; CT indicates computer tomograph; LA, left atrium; PA, pulmonary artery

**Fig. 2:**
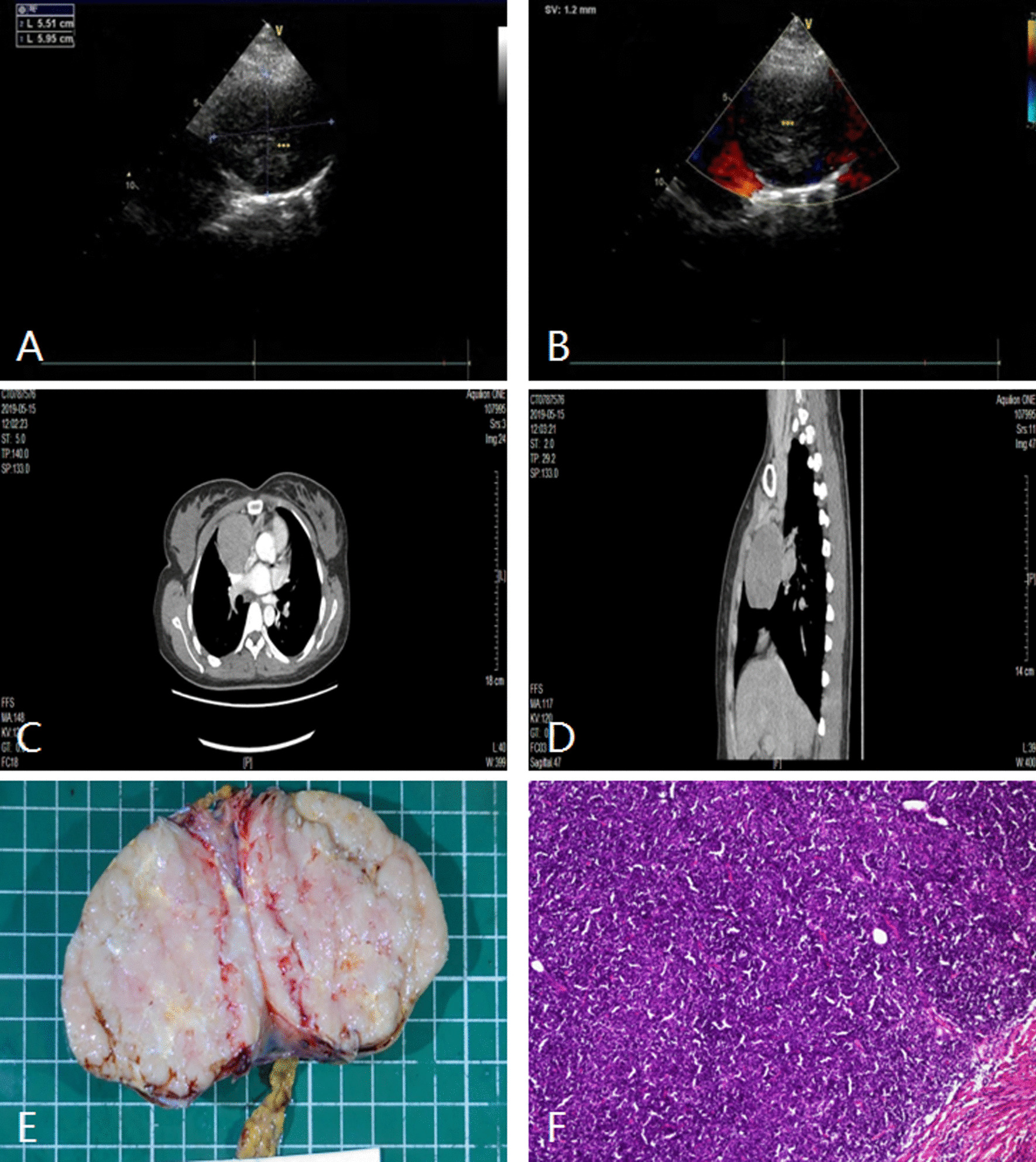
Anterior mediastinal mass (thymoma) in a 30-year-old woman. **A** and **B** TTE showed a well-circumscribed round mass in the anterior mediastinum. **C** and **D** Contrast-enhanced CT showed an regularly shaped mass with the uniform density and the moderate enhancement. **E** and **F** The pathologic diagnosis of this mass was type AB thymoma. The gross specimen shows an intact capsule and a grayish cut surface. TTE indicates transthoracic echocardiography; CT, computer tomograph; asterisks, mass

**Fig. 3:**
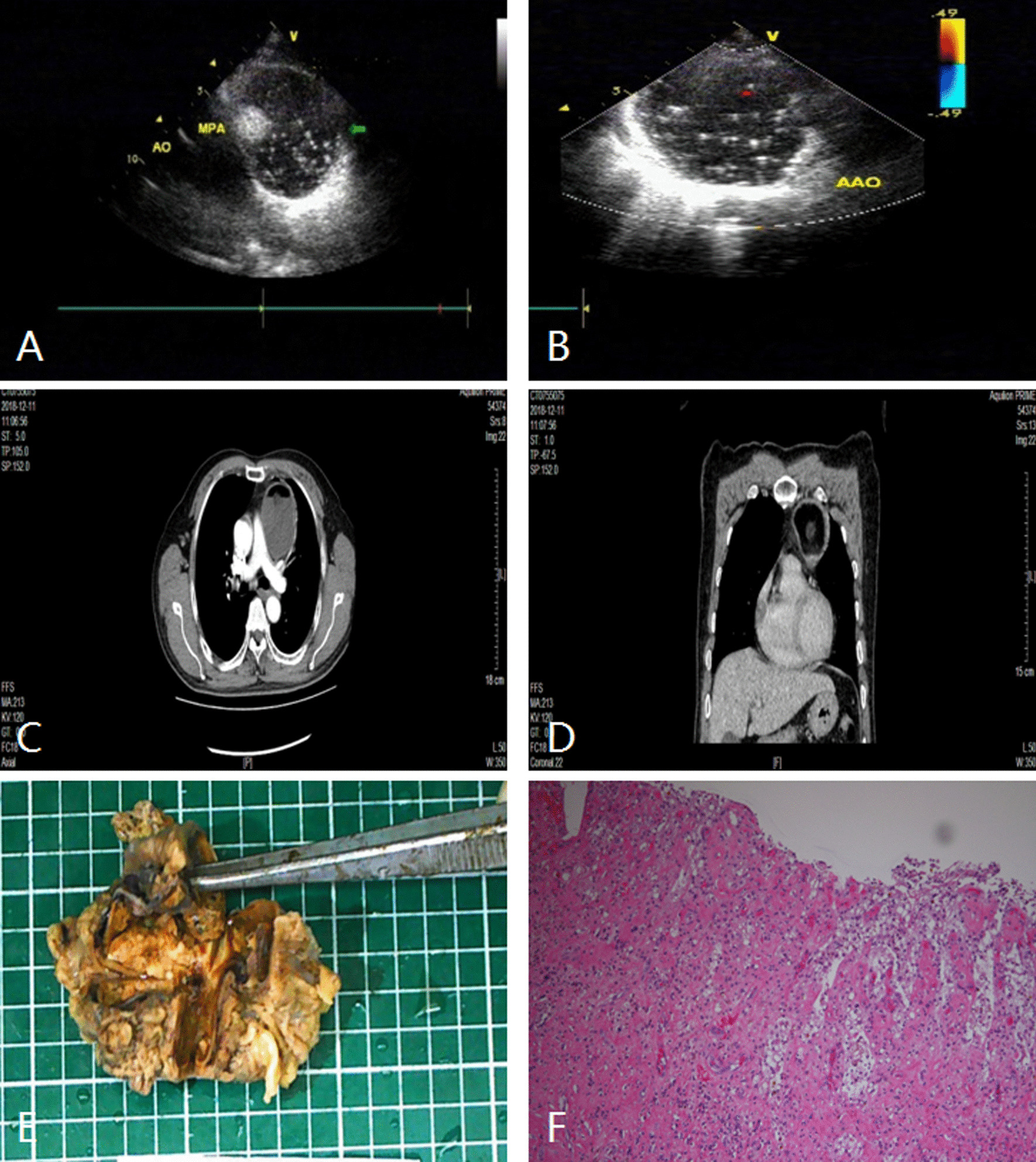
Anterior mediastinal mass (teratoma) in a 59-year-old man. **A** and **B** TTE showed a well-circumscribed round cystic-solid mass in front of the aorta and pulmonary artery. **C** and **D** Contrast-enhanced CT showed a round like abnormal density shadow with smooth boundary, with liquid and fat density shadow in it, presenting as fat liquid plane, with slightly thick wall and annular calcification and no obvious enhancement. **E** and **F** The pathologic diagnosis of this mass was mature cystic teratoma. The wall of the capsule is thick and the structure of bean curd residue can be seen in it. TTE indicates transthoracic echocardiography; CT indicates computer tomograph; AO indicates aorta; AAO indicates ascending aorta; PA, pulmonary artery

**Fig. 4:**
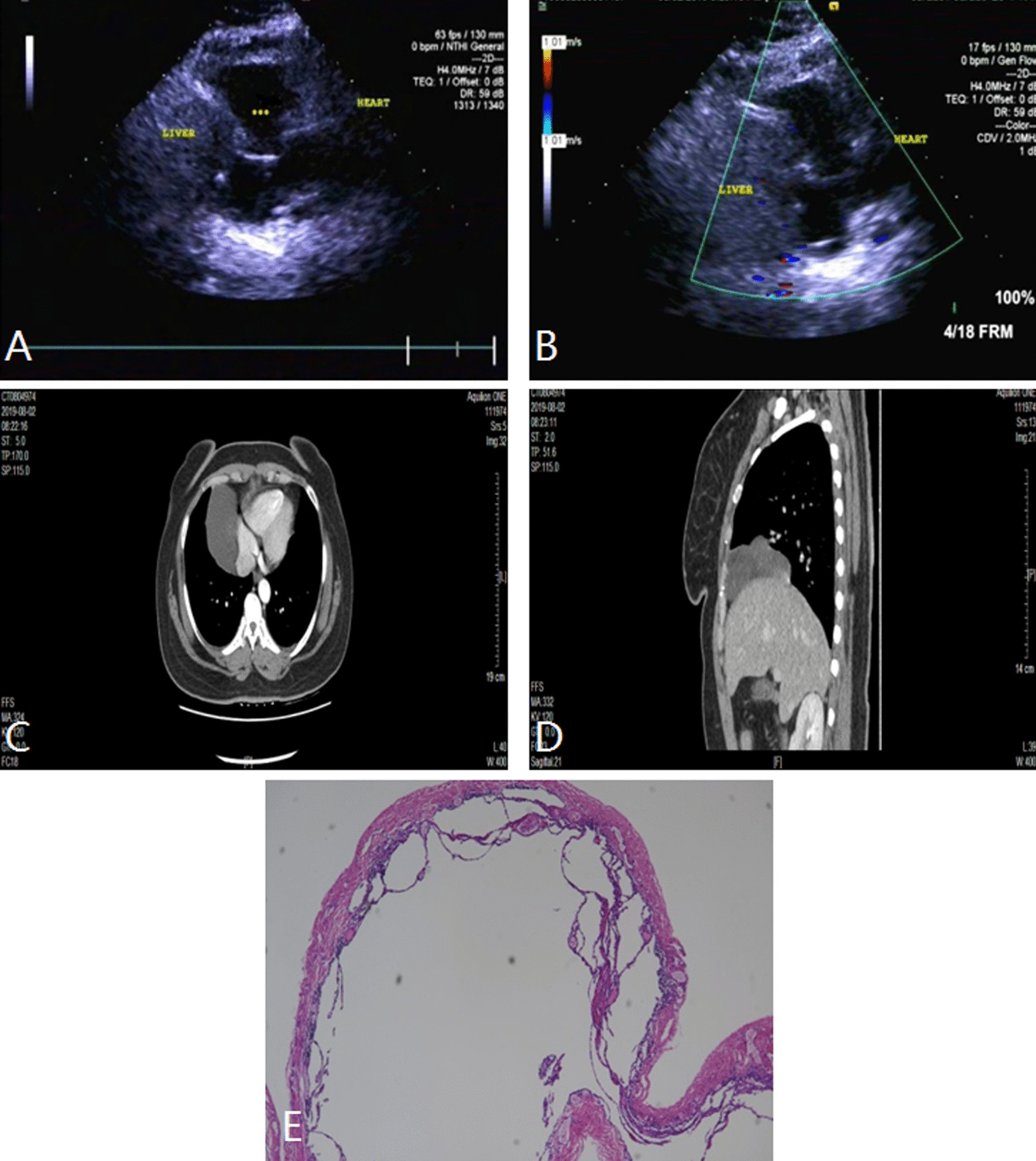
Middle mediastinal mass (pericardial cyst) in a 41-year-old woman. **A** and **B** TTE showed a well-circumscribed anechoic mass between the heart and the liver. **C** and **D** Contrast-enhanced CT showed a regularly shaped mass with the cystic hypodensity and no enhancement. **E** The pathologic diagnosis of this mass was pericardial cyst. The wall of the cyst was lined with cuboidal epithelium, and the cavity was filled with clear cystic fluid. TTE indicates transthoracic echocardiography; CT, computer tomograph; asterisks, mass

The maximum diameter of the lesions ranged from 3.2 cm to 13.66 cm, the mean diameter was about 5.6 ± 1.83 cm, and the median diameter was about 7.4 cm, among which 29 cases (29/49,59.18%) were larger than 5 cm. 42 cases (42/49, 85.71%) were seen in the parasternal and apex views, 3 cases (3/49, 6.12%) in the parasternal and subxiphoid views, and 5 cases (5/49, 10.20%) in the supra sternal fossa view (see Table 2 for details). There were 3 types of echo: cystic anechoic lesions in 4 cases (4/49, 8.16%) were pericardial cyst, cystic-solid echogenic lesions in 2 cases (2/49, 4.08%) were teratoma, and hypoechoic or heterogeneous echoing lesions in the remaining 43 cases (43/49, 87.76%) were solid tumors (see Table 2 for details, Figs. 1, 2, 3, 4). 6 cases compressed the heart and 21 cases encroached on the heart and vessels.

**Table 2:** The distribution and ultrasonic informations of extracardial lesions

	N	%	Disease type	Ultrasound view	Specific location	Echogenicity
Lung lesions	8	16.33	Central lung cancer	Parasternal view	Pulmonary artery bifurcates near the left hilum	Hypoechoic or heterogeneous echo
			Peripheral lung cancer	Supra sternal fossa view	Lateral to the descending aorta	Hypoechoic or heterogeneous echo
			Metastatic sarcoma of the lung	Parasternal view	Behind the left atrial	Hypoechoic or heterogeneous echo
Lymph node lesions	14	28.57	Mediastinal lymph node metastasis	Parasternal view	Lateral to pulmonary artery	hypoechoic
			Lymphoma	Parasternal view	Pericardial sinus and surrounds the heart	Hypoechoic
Pericardial lesions	5	10.20	Pericardial cyst	Subxiphoid view	Lateral to right heart	Cystic echoless
			Pericardial mesothelioma	Subxiphoid view	Next to the superior vena cava	Heterogeneous echo
Esophageal lesions	15	30.61	Esophageal cancer	Parasternal view apex view	Behind the left atrial	Hypoechoic or heterogeneous echo
			Hiatal hernia	Parasternal view apex view	Behind the left atrial	Heterogeneous echo
Others	7	14.29	Teratoma	Parasternal view	In front of the heart	Cystic-solid echo
			Thymoma	Parasternal view	In front of the heart	Hypoechoic
			Lipoma	Parasternal view	Lateral to pulmonary artery	Heterogeneous echo

Diagnosis coincidence rates of ultrasonic and CT were respectively 77.55% and 93.88%, with a statistical difference (see Table 3 for details).

**Table 3:** Comparison of coincidence rate between ultrasound and CT in diagnosing extracardiac lesions

	Right	Wrong	Diagnostic coincidence rate (%)	χ^2^ value	P value
TTE	38	11	77.55	6.308	0.012
CT	46	3	93.88		

TTE indicates transthoracic echocardiography; CT indicates computer tomograph

## Discussion

The normal cardiac is located in the middle mediastinum and surrounded by pericardium, lung, pleura, trachea, esophagus, diaphragm, lymph, nerve, fat and other tissues. The value of TTE in the detection of myocardial and intra-cardiac lesions is clear and affirmative, on the contrary, the abnormal extra-cardiac lesions are often unrecognized.

In this study, 37 cases (37/49, 75.51%) of extra-cardiac lesions were malignant lesions, mainly including lung cancer, esophageal cancer, lymphoma and mediastinal lymph node metastasis. 12 cases (12/49, 24.49%) were benign lesions, mainly including pericardial cyst and teratoma. Most of abnormal extra-cardiac space occupying lesions in our group were relatively larger, and the maximum diameter range was 3.2 cm–13.66 cm, of which 29 cases (29/49, 59.18%) were larger than 5 cm. It is speculated that the larger lesions may be more sensitively detected by TTE.

### Lung lesions

The trachea and hilum of the lung are located in the mediastinum adjacent to the cardiac, and these structures are not clearly displayed by TTE in general conditions. However, solid or cystic masses that appear in the surrounding tissue adjacent to the heart can be detected by TTE. 8 cases (8/49, 16.33%) of lung lesions in this group were all malignant, which were consist of 3 cases of primary central lung cancer, 4 cases of left or right upper lung cancer and 1 case of sarcoma pulmonary metastasis. Ultrasound manifestations of sarcoma pulmonary metastasis and central lung cancer were hypoechoic solid masses in the hilar region adjacent to the heart (behind the left atrium or beside the pulmonary artery/vein) on sonography, which were seen in the parasternal and apex views (Fig. 1). Another 4 cases of primary left or right upper lung cancer presented as hypoechoic solid masses near the descending aorta or superior vena cava in the view of supra sternal fossa.

In an autopsy study of 100 cases of lung cancer patients, Nichols et al. [4] found that 19% of the deaths of lung cancer patients were related to cardiac involvement, such as bloody pericardial effusion caused by pericardial metastasis, pulmonary embolism caused by pulmonary artery cancer thrombus, etc. The heart involvement can be found by TTE in time. In this group, 3 cases of primary central lung cancer encroached on the heart, including 1 case of pericardium involving, 1 case of left pulmonary artery involving and 1 case of right lower pulmonary artery and pulmonary vein involving. It was inferred that large central lung cancer in the hilar region can be observed in the parasternal and apical views, furthermore, the heart is more vulnerable to invasion. On the contrary, the lesions in the upper lung detected in the suprasternal fossa view were so far away from the cardiac that is not easy to be affected.

### Mediastinal lesions

Mediastinum is the general term of the organs, structures and connective tissues between the left and right pleura. In general, it is divided into anterior mediastinum, middle mediastinum and posterior mediastinum by pericardium.

Mediastinal ultrasound [5–12] was mentioned more than 30 years ago, but has been rarely used in clinical routine in china. The mediastinum is surrounded by thoracic and lung tissues which are not conducive to the penetration of acoustic beam. The fluid dark areas (blood) in atria, ventricles and large vessels can be as an acoustic window to show abnormal lesions in surrounding tissues of cardiac in TTE examination. In this group, mediastinal lesions were predominant, with a high proportion of 83.67% (41/49), among which 31 cases (31/41, 75.61%) were malignant. Mediastinal tumors are often accidentally found in conventional TTE.

### Anterior mediastinal lesions

Thymoma, teratoma and lymphoma are common tumors in the anterior mediastinum [13–15]. Due to large size and protruding to the outer edge of the sternum, these lesions are easy to be detected by TTE. There were 12 cases of anterior mediastinal masses that accounted for 29.27% of all mediastinal lesions (see Table 1 for details). Anterior mediastinal masses are mostly located anterior to the right ventricle and the ascending aorta or pulmonary artery. Benign thymoma in 2 cases presented as a round or quasi round solid mass with clear boundary and homogeneous hypoechoic inside on ultrasonographic (Fig. 2). 1 case of invasive thymoma was found as a unsmooth capsule, unclear boundary and different internal echoic intensity. Teratoma in 3 cases presented as a cystic-solid echo mass with clear boundary and various internal echoes (Fig. 3), which could be shown as alternating internal strong, high, low and medium echo areas, similar to the common ovarian teratoma. This phenomenon is attributed to the complex internal structure of teratoma, with fat, fluid, soft tissue, calcification, etc. Anterior mediastinum is the most common site of intra-thoracic lymphoma as well. There were 6 cases of lymphoma located in the anterior mediastinum in this group, which showed round, oval, lobulated or irregular hypoechoic masses with uneven internal echo and infiltrative growth around on ultrasonography.

### Middle mediastinal lesions

Pericardial space occupying lesions are generally located in the middle mediastinum outside the cardiac contour. Ultrasound is superior to other imaging examinations in detecting cystic lesions because of high sensitivity. Cystic lesions in the mediastinum can be of various origins, including bronchogenic cysts, pericardial cysts, thymic cysts, cystic teratoma, and lymphangioma enhancement, most of which are benign. In this study, there were 2 cases of pericardial cysts located in the right anterior diaphragmatic angle of the heart. The sonogram of pericardial cysts showed quasi round or irregular anechoic area, with thin and smooth wall, and no communication with the cardiac cavity (Fig. 4). Cho Jeong et al. [16] reported a giant pericardial cyst with cardiac tamponade. In our group, 2 cases of pericardial cysts only compressed right heart a little and no obvious tampomade. Malignant pericardial lesions are rare, most of which are pericardial metastatic tumors or primary malignant pericardial mesothelioma. In our group, 1 case of malignant pericardial mesothelioma was located in the anterolateral pericardial cavity of the right atrium, which presented as a heterogeneous hypoechoic mass with irregular shape and calcification. In addition, invasion of the right atrium and slight pericardial effusion were observed as well in this case. Lymph node lesion is an another common lesion in the middle mediastinum, including 2 cases mediastinal lymph node metastasis and 3 cases of lymphoma in this study. The sonogram of mediastinal lymph node metastasis showed a hypoechoic mass with clear boundary, which was the fusion of several enlarged lymph nodes. 3 cases of lymphoma in the middle mediastinum was similar to that in the anterior mediastinum on ultrasonography.

### Posterior mediastinal lesions

**Fig. 5:**
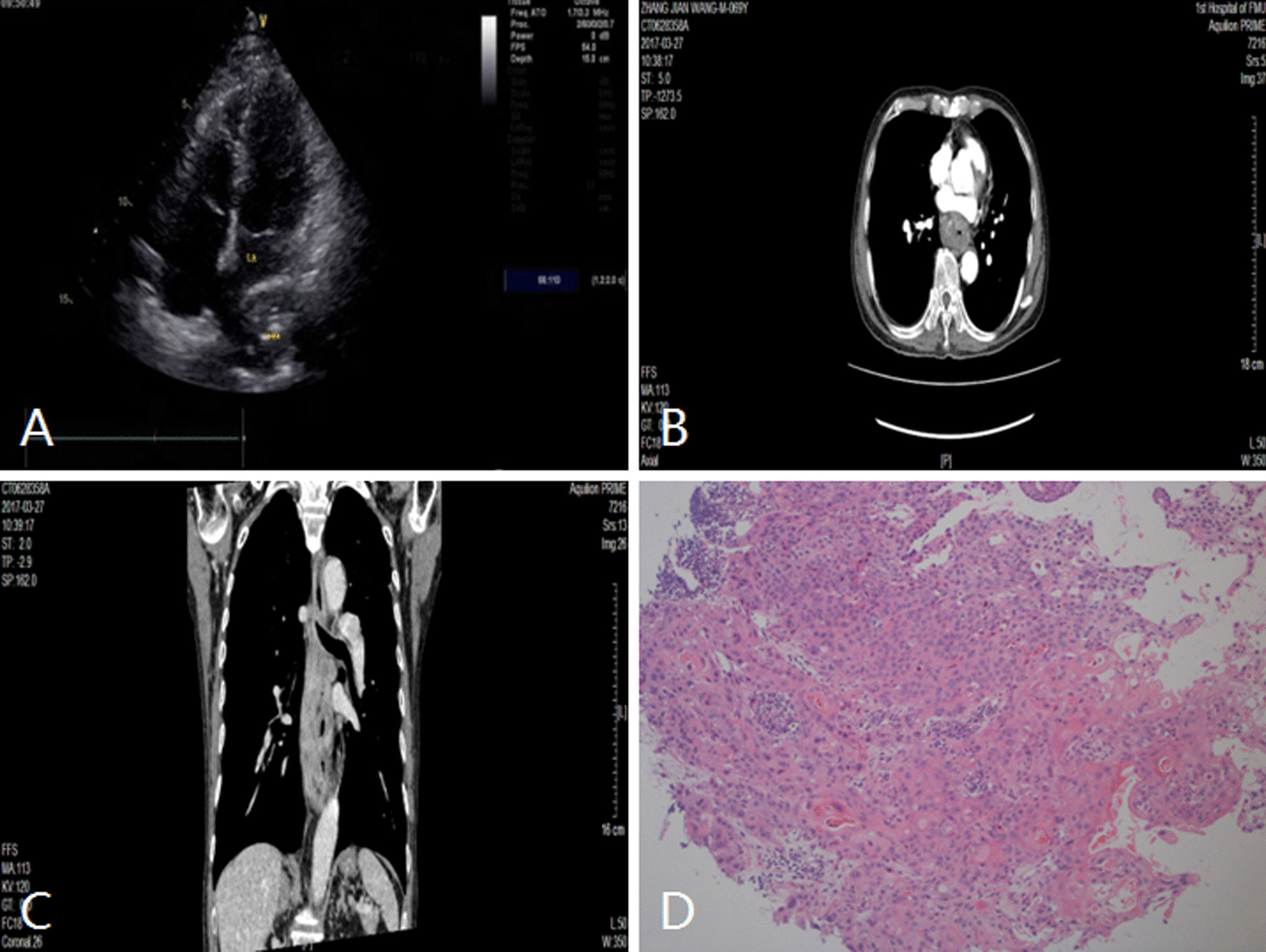
Posterior mediastinal mass (esophageal cancer) in a 69-year-old man. **A** TTE showed a round hypoechoic or heterogeneous echo mass with hypoechoic periphery and irregular hyperechoic interior, similar to “pseudo kidney” shape. **B** and **C** Contrast-enhanced CT showed a regularly shaped mass with inhomogeneous low-density shadow, obvious inhomogeneous enhancement behind left atrium. The esophageal wall was diffusely and irregularly thickened, and the corresponding lumen was irregularly narrow. **D** The pathologic diagnosis of this mass was esophageal squamous cell carcinoma. TTE indicates transthoracic echocardiography; asterisks, mass; LA, left atrium

There were 15 cases of esophageal lesions and 3 cases of metastatic lymph nodes in the posterior mediastinum. The ultrasonographic findings of post mediastinal metastatic lymph nodes were consistent with the above. The esophageal cancer (14/15, 93.33%) accounted for the majority of posterior mediastinal lesions in this study. The lesions of upper and middle esophagus are unable to be detected due to the interference of the field of vision of lung gas [17]. For this reason, all of 14 cases of esophageal cancer detected by TTE were located in the middle and lower esophageal segment, which were confirmed by CT. Esophageal carcinoma presented as a round hypoechoic or heterogeneous echo mass in the posterior top of the left atriumin in the parasternal and apical views, with hypoechoic periphery and irregular hyperechoic interior, similar to “pseudo kidney” shape, separating the left atrium from the thoracic aorta (Fig. 5).

None of neurogenic tumor was detected in this study. TTE is not easy to detect small deep tumors in the posterior mediastinum due to the interference of lung gas. In addition, some studies [18–21] found that contrast-enhanced ultrasound can improve the targeting of fine-needle aspiration and identify the nature of mediastinal tumors.

## Conclusion

In conclusion, TTE could provide useful information of the location, size, echogenicity, and relationships with the heart and the cardiac vessels. Although the diagnostic coincidence rate of TTE was slightly lower than that of CT, TTE has certain diagnostic value for extra-cardiac lesions. The sonographer should improve the awareness of extra-cardiac lesions through multi-slice scanning, especially the non-standard section.

## Data Availability

All data generated or analyzed during the present study are included in this article.

## Abbreviations

TTE: Transthoracic echocardiography
CT: Computed tomography
MRI: Magnetic resonance imaging
